# Cardiac Arrest Due to Air Embolism: Complicating Image-guided Lung Biopsy

**DOI:** 10.7759/cureus.3295

**Published:** 2018-09-13

**Authors:** Zaineb Viqas, Allah Yar, Maria Yaseen, Muhammad Khalid

**Affiliations:** 1 Medicine, Fatima Jinnah Medical University, Lahore, PAK; 2 Internal Medicine, Sandeman Provincial Hospital, Quetta, PAK; 3 Internal Medicine, Dow University of Health Sciences, Karachi, PAK; 4 Department of Internal Medicine, East Tennessee State University, Quillen College of Medicine, Johnson City, USA

**Keywords:** cardiac arrest, air embolism, lung cancer, pulmonary nodule, left ventricular air embolus

## Abstract

Cardiac arrest due to air embolism is an infrequent complication. Air embolism can be associated with procedures like endoscopic retrograde cholangiopancreatography, endoscopic variceal ligation, operative hysteroscopy, laparoscopic surgery, pacemaker placement, cardiac ablation, fiberoptic bronchoscopy, and decompression sickness. In rare cases, air embolus can be a catastrophic complication of computed tomography (CT) guided lung biopsy, which can lead to cardiac arrest. We present a case of a 67-year-old male chronic smoker with a left lower lobe pulmonary nodule who had a cardiac arrest due to air embolism as a consequence of CT guided biopsy of the pulmonary nodule found on a CT scan of the chest. He was successfully resuscitated and intubated for mechanical ventilation. He was managed conservatively and discharged home in a stable condition.

## Introduction

Air embolism is an uncommon but potentially catastrophic event that occurs as a consequence of entry of air into the vasculature. Surgery including laparoscopic surgery, vascular interventions, trauma, deep sea diving, and barotrauma from mechanical ventilation are the commonly known causes of air embolism [1-4]. We present a case of cardiac arrest from air embolism in the left ventricle following fine needle lung biopsy.

## Case presentation

**Figure 1:**
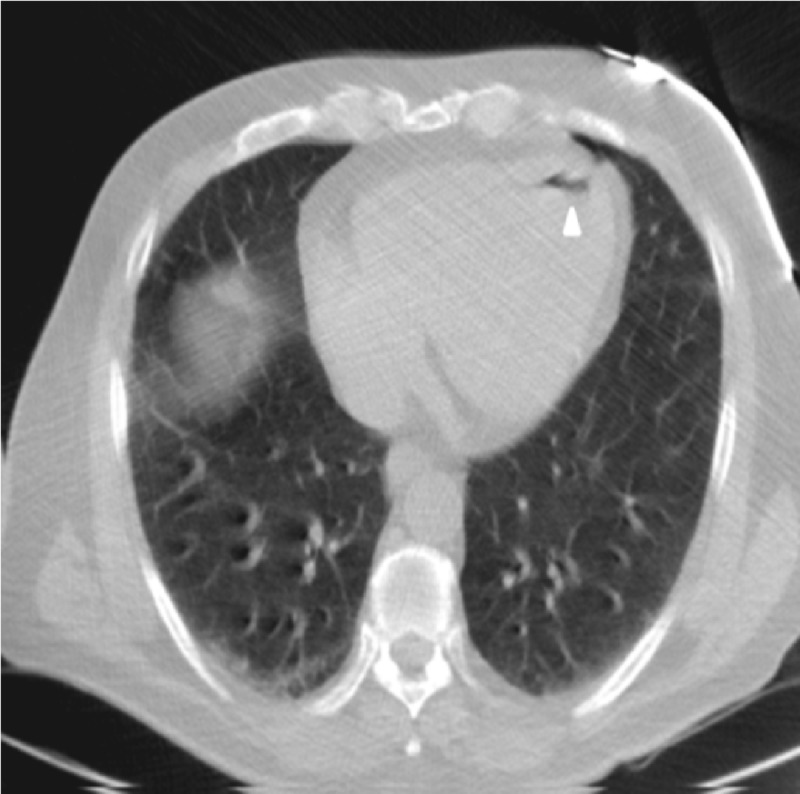
Computed tomography scan of the chest showing air in the left ventricle (arrowhead).

A 67-year-old male chronic smoker with medical history significant for chronic obstructive pulmonary disease was admitted for imaging guided biopsy of a 1.2 cm left lower lobe lung nodule found recently on a computed tomography (CT) scan of the chest. The nodule was highly suspicious for primary lung malignancy. The patient was placed in a prone position and lung parenchyma in the posterior lateral left chest was visualized. Under CT guidance, a 19-gauge guide was advanced into the left lower lobe and two separate 20-gauge core biopsy specimens were obtained from the mass. There was no hemorrhage or immediate post procedure complication. However, towards the end of the procedure, the patient started complaining of sudden onset of chest pain and became unconscious. No palpable pulses were identified. A code blue was called and cardiopulmonary resuscitation was begun according to Advanced Cardiovascular Life Support guidelines. The patient subsequently demonstrated ventricular fibrillation which responded to defibrillation shock and epinephrine. The patient had three cycles of chest compressions, one dose of epinephrine, and a shock of 200 J. He was successfully resuscitated and intubated for mechanical ventilation. A CT scan of the chest was obtained immediately after the resuscitation and it demonstrated development of a small anechoic area in the left cardiac ventricle consistent with air embolus (Figure 1).

The vital signs recorded at the time were as follows: blood pressure of 130/80 mmHg, pulse rate of 90 beats per minute, respiratory rate of 18 breaths per minute, and normal oxygen saturation of 92% on room air. The patient was admitted to the intensive care unit. Bedside transthoracic echocardiogram (TTE) did not reveal evidence of an air embolus. Additionally, no cardiac wall motion abnormalities were noted. The patient remained hemodynamically stable for 24 hours, and he was successfully weaned off from the mechanical ventilator next day and discharged home in a stable condition.

## Discussion

Air embolism is an uncommon but potentially catastrophic event that occurs as a consequence of entry of air into the vasculature. It can be venous or arterial. Venous air embolism occurs when air enters the systemic venous circulation and travels to the right ventricle and pulmonary circulation. Arterial air embolism occurs when air enters the systemic arterial circulation, which can cause ischemia of the systemic organs. The different possible mechanisms of air embolism include direct communication between the source of air and the vasculature, for example, a broncho-arterial fistula, or it can be a pressure gradient favoring the passage of air into the circulation like in ear, nose, throat surgeries, and in neurosurgical procedures when the incision is superior to the heart at a distance that is greater than the central venous pressure. The negative venous pressure relative to the atmosphere favors the passage of air into the circulation, especially when the patient is in a sitting position [5].

Computed tomography (CT) guided transthoracic needle biopsy is a frequently performed procedure by interventional radiology for evaluation of pulmonary nodules because of its high diagnostic accuracy [6]. The common complications related to this procedure are pneumothorax (10 to 17%) and hemorrhage assessed clinically as hemoptysis (1 to 6.9%) [7,8]. Systemic air embolism during CT guided lung biopsy is a rare complication with reported incidence ranging from only 0.02% to 0.06% [9,10]. It can be easily missed most of the times, which could result in fatal consequences. Different risk factors that determine the risk of cardiac embolism include large size of biopsy needle, location of pulmonary lesions especially in the lower lobe, occurrence of parenchymal hemorrhage [11], and the consistency and type of lesion whether it is bullous or cystic.

The clinical symptoms depend upon the amount of air entering the circulation. Usually it is asymptomatic and self-limiting with small amount of air entry into the vasculature. A large amount of air would clinically manifest itself. Cardiac air embolism would typically present with symptoms of chest pain and shortness of breath with potential of lodging in the brain, which presents with altered mental status, dizziness, lightheadedness, and focal neurological deficits. The classic signs of cardiac air embolism include tachycardia, bradycardia, hypotension, crackles, wheezing, tachypnea, jugular venous distension, and a water-wheel or mill-wheel murmur, which is a splashing sound due to the presence of gas in the cardiac chamber. It can also manifest as shock, hypoxemic respiratory failure, and cardiac arrest.

It can be avoided by careful selection of insertion site with less exposure to lung parenchyma, minimizing entry of external air to pulmonary veins, instructing the patient to avoid breathing deeply and avoiding cough and straining during the course of the procedure, occluding the introducer needle by the inner stylet, saline drops, or the operator’s finger, and avoiding a kink in the coaxial biopsy system [12].

Diagnosis depends upon high clinical suspicion and imaging modalities. Laboratory results can show thrombocytopenia and elevated cardiac enzyme. Electrocardiogram can show findings of acute ischemia or myocardial infarction. Arterial blood gas panel may indicate hypoxemic or hypercapnic respiratory failure. Chest X-ray could be normal or it may demonstrate atelectasis or intracardiac air. In case of massive air embolism, ventilation perfusion (VQ) scan could show VQ mismatch. CT of the chest can show air embolism in the central veins, right ventricle, pulmonary artery, or heart. Echocardiography could sometimes be used to rapidly identify air in the cardiac chambers or great veins, right ventricular dilatation, or pulmonary hypertension.

Treatment includes airway support, volume resuscitation, vasopressors, intubation with mechanical ventilation, hyperbaric oxygen chamber therapy, withdrawal of air from the cardiac chamber usually from the right heart with a Swan-Ganz catheter, close chest cardiac massage, and putting the patient in left lateral decubitus and/or Trendelenburg position. Another recommendation is a head down position. However, in our case, the patient was placed in a supine position because that is less prone to cause cerebral edema [13-15]. Early identification and treatment of this condition is the key to prevention of fatal consequences.

To our knowledge, only a few cases of fatal cardiac arrest complicating transthoracic lung biopsy have been reported in the literature. Most of the times, these patients had multiple cancer metastases, chronic diseases, or had hemoptysis or cough during the needle biopsy, which could have led to cardiac air embolism [16-20]. Nevertheless, we think that cardiac air embolism during CT guided lung biopsy is a rare adverse event, but its true incidence is probably underestimated.

## Conclusions

CT guided transthoracic needle biopsy is a resourceful and widely used tool to evaluate pulmonary nodules. Cardiac air embolism is one of its rare but potentially fatal complications. Due to its life-threatening nature, early identification and treatment requires prudent clinical judgement. When performing CT guided transthoracic needle biopsy, nursing staff and physicians should be aware of the risk factors, classic clinical signs and symptoms. Prompt emergency treatment is with airway support, volume resuscitation, vasopressors, intubation with mechanical ventilation, and hyperbaric oxygen chamber therapy. Adequate management can save lives.
